# Incorporating Personality Traits to Assess the Risk Level of Aberrant Driving Behaviors for Truck Drivers

**DOI:** 10.3390/ijerph18094601

**Published:** 2021-04-26

**Authors:** Chien-Hung Wei, Ying Lee, Yu-Wen Luo, Jyun-Jie Lu

**Affiliations:** 1 Department of Transportation and Communication Management Science, National Cheng Kung University, Tainan City 701, Taiwan; louiswei@mail.ncku.edu.tw (C.-H.W.); may25357418@gmail.com (Y.-W.L.); ji432598@gmail.com (J.-J.L.); 2 Department of Supply Chain Management, National Kaohsiung University of Science and Technology, Kaohsiung 811, Taiwan

**Keywords:** truck drivers, personality traits, aberrant driving behavior, artificial neural network, driving risk level

## Abstract

Economic globalization and the internet economy have resulted in a dramatic increase in freight transportation. Traffic crashes involving trucks usually result in severe losses and casualties. The fatality and injury rates for heavy truck accidents have been 10 times higher than for sedans in Taiwan in recent years. Thus, understanding driving behavior and risk are important for freight carriers. Since personality traits may result in different driving behaviors, the main objective of this study is to apply artificial neural network (ANN) models to predict the frequency of aberrant driving behavior and the risk level of each driver according to drivers’ personality traits. In this case study, relevant information on truck drivers’ personality traits and their tendency to engage in aberrant driving behavior are collected by using respectively a questionnaire and a fleet surveillance system from a truck company. A relative risk level evaluation mechanism is developed considering the frequency and distribution of aberrant driving behavior. The Jenks natural breaks optimization method and the elbow method are adopted to optimally classify 40 truck drivers into 4 aberrant driving behavior levels and 5 driving risk levels. It was found that 5% of drivers were at the highest aberrant driving behavior level, and 7.5% of drivers were at the highest driving risk level. Based on the results, the proposed models show good and stable predictive performance, especially for the class of drivers with excessive rotation speed, hard acceleration, excessive rotation speed, hard deceleration, and driving risk. With the proposed models, the predictive class for aberrant driving behavior and driving risk can be determined by plugging in a driver’s personality traits before or after employment. Based on the prediction results, the manager of a transportation company could plan the training program for each driver to reduce the aberrant driving behavior occurrence.

## 1. Introduction

Trucks are typical commercial delivery vehicles that can be subdivided into two categories: Light and heavy trucks. Table 1 shows statistics on traffic crashes in Taiwan for 2019 [1]. Although the number of crashes, fatalities, and injuries caused by sedans is relatively high, the crash, fatality, and injury rates of heavy trucks are 10 times higher than those of sedans. This shows that truck safety is an urgent issue in Taiwan.

For instance, most truck drivers are male, work long hours with tight schedules, spend more time in road traffic, and are on average older than other drivers [2,3]. Therefore, truck drivers are more likely to have high fatality rates and injury risks in crashes than other drivers. Among all possible factors causing traffic crashes, aberrant driving behavior is usually regarded as the most significant [4]. Among different groups of drivers, truck drivers have certain personality traits and driving behaviors that may increase their risk of causing a traffic accident. If the relationship between the drivers’ frequency of aberrant driving behavior and the drivers’ personality traits can be conducted, the manager of a transportation company can refer the drivers’ personality traits to predict drivers’ aberrant driving behavior and plan the training program for each driver. Furthermore, the transportation company can reduce the probability of traffic crashes and further raise corporate integrity and reputation.

Therefore, this study aims to apply the artificial neural network (ANN) technique to explore a relational model between personality traits and aberrant driving behavior. The personality traits considered in this study are extraversion, agreeableness, conscientiousness, neuroticism, and openness to experience. The data were collected from 40 drivers employed by a truck company. The drivers’ aberrant driving behaviors were recorded by a digital tachograph, including behaviors such as exceeding the speed limit, abnormal stay (slow driving with limited range), hard acceleration and deceleration, driving overtime, and excessive rotation speed. Data analysis is addressed in Section 3. Because the frequencies of aberrant driving behavior among drivers are different, it is difficult to directly use these raw data for model development. Before modeling, this study adopted the Jenks natural breaks optimization method and the elbow method to classify the frequencies of aberrant driving behaviors and driving risk index into levels, as shown in Section 4. The ANN model development and the evaluation of prediction results are presented in Section 5. Section 6 provides a sensitivity analysis for the features of the proposed models. Finally, Section 7 presents concluding remarks. To evaluate drivers’ performance and decrease the occurrence of traffic crashes, the administrative manager of a truck company could use this validated model to plug in human factor data and predict driving behavior.

## 2. Literature Review

Driving safety has been widely discussed in the literature. Previous studies have usually focused on the relationship between various factors and driving behavior. In this section, related reports on personality traits and driving behavior are reviewed. In the following, Section 2.1 reviews the categories and influence of aberrant driving behavior, and Section 2.2 covers the personality traits.

### 2.1. Aberrant Driving Behaviors

The aberrant driving behaviors can be measured from Driver Behavior Questionnaire (DBQ), traffic sanctions, or vehicle-mounted sensors. The researches that apply the data from vehicle-mounted sensors mostly focus on conducting the algorithm or model to detect or recognize [5,6,7] the aberrant driving behaviors. Because the vehicle-mounted sensor is not very popular and costly, few researchers explore the influences to aberrant driving behaviors using vehicle-mounted sensors to collect the data. Some researchers use self-reported traffic sanctions as the drivers’ aberrant driving behaviors [8]. DBQ is a self-reported method and one of the most widely used instruments to measure drivers’ risky behaviors and bad habits in daily driving [2,9,10,11,12,13,14,15]. However, the self-reported method may result in biased data.

Previous studies showed that job strain [16], low social support at work [16], more driving hours [12], less driving experience [12,17], younger drivers [2], and errors in behavioral inhibition [15] were positively and significantly associated with risky driving behavior.

In addition, to explore the significant factors affecting aberrant driving behavior, some studies prove that aberrant driving behaviors [2,12,14] are significantly related to traffic crashes or accident risk, even for different countries or research subjects.

To explore the relationship between the selected variables and aberrant driving behaviors, Structural Equation Models (SEM) [14,16] and logistic regression model [2,11,12] are commonly used methods.

Professional drivers spent more time driving than others, and more attention should be paid to their driving behavior. Thus, previous studies have focused on taxi drivers [12], bus drivers [14,16], and truck drivers [2,17] as research objects.

### 2.2. Personality Traits

In addition, to discuss the impact on aberrant driving behavior from physiology, socioeconomic and socio-cognitive factors, previous studies have investigated the potential relationship between drivers’ personality traits and driving behavior. The Big Five personality traits developed from the 1980s onwards in psychological trait theory included extraversion, agreeableness, conscientiousness, neuroticism, and openness to experience.

Dahlen and White [18] incorporate the Big Five factors (personality traits), sensation seeking, and driving anger to predict unsafe driving. Their study results found that openness, emotional stability, agreeableness, trait driving anger, and sensation seeking are significant in the prediction of driving behavior. Extraversion and conscientiousness did not appear useful in understanding driving behavior.

Shen et al. [19] explored the relationship between positive driving behavior and the Big Five personality traits for 421 drivers in China. Different from previous study results, their research shows that neuroticism was negatively associated with positive driver behaviors.

Mallia et al. [14] collected 301 bus drivers’ data and applied SEM to prove personality–attitudes associated with aberrant driving behaviors and violations associated with crash risk.

Li’s [20] research forecasted aberrant driving behavior using the Big Five personality traits. The study constructed levels of driving risk by relating aberrant driving behavior to driving risk. The five personality traits were used to predict an individual driver’s risk level by ordered logit regression. The results showed that drivers with more neurotic or conscientious traits could be classified as higher risk, and more agreeable drivers could be classified as lower risk.

Based on the results of the literature review, the influence on aberrant driving behavior from extraversion, agreeableness, conscientiousness, neuroticism, and openness to experience may not be consistent from different study areas or research objects.

## 3. Model Building

### 3.1. Data Collection

The data were collected from 40 drivers employed by a truck company. All drivers were male. Eleven drivers were younger than 40 years old. Fourteen drivers were between 40 and 49 years old. Fifteen drivers were older than 49 years old. Twelve drivers’ driving experience was less than 11 years. Fifteen drivers’ experience was between 11 and 20 years. Thirteen drivers’ experience was more than 20 years.

#### 3.1.1. Personality Traits

Previous studies have shown that personality can have a considerable impact on driving behaviors or driving risk [14,18,19,20]. Although there were differences in the classification and definition of personality, most of these studies have found that personality was related to driving operations. This section explores our approach for collecting personality data for the study subjects using a questionnaire. The measurement dimensions and questions in our personality questionnaire were based on the Big Five personality classification proposed by Costa and McCare [21]. The personality traits considered in this study were extraversion, agreeableness, conscientiousness, neuroticism, and openness to experience. We based our questionnaire on those developed by Chen [22]; the questions and answers, measured by a five-point Likert scale, are presented in Table 2. The data were collected from 40 drivers employed by a truck company. When the driver attended the monthly traffic safety meeting, the driver was invited to complete this questionnaire survey individually.

#### 3.1.2. Aberrant Driving Behavior

Differed from the previous research using self-reported data to collect aberrant driving behavior, this study aims to collect the behavioral data of truck drivers using vehicular-mounted that assessed driving performance. The drivers’ aberrant driving behaviors include exceeding the speed limit, abnormal stay, hard acceleration and deceleration, driving overtime, and excessive rotation speed. The Tachograph on-board sends digital data of the drivers’ aberrant driving behaviors every 30 seconds via wireless communication module to a fleeting monitoring center. The trucks usually drive on intercity highways and urban arterials. To analyze the data, we used the following approach. The statistical results of 40 drivers’ aberrant driving behaviors during a period of three months are summarized in Table 3. Based on these results, we found wide differences in aberrant driving behaviors among drivers, making the direct application of data impractical for model development. Therefore, we needed to classify the drivers’ aberrant behaviors into subgroups, which can also be referred to as risk levels. The classification criteria for each driving behavior measure were the following: (1) Exceeding speed limit: The speed was greater than 87 km/h for more than 90 s; (2) abnormal stay: The driving speed was lower than 5 km/h, with a movement range less than 50 m and a duration of more than 5 min; (3) hard acceleration: The difference in speed was greater than 30 km/h between the first second and fifth seconds; (4) hard deceleration: Vehicle speed reduced by more than 13 km/h per second for three consecutive seconds; (5) driving overtime: The vehicle’s continuous driving time, which exceeds the maximum time limit defined by the company’s policy maximum limit; (6) excessive rotation speed: The speed was less than 85 km/h, while the rotation was more than 1500 rpm for three consecutive seconds.

**Figure 1:**
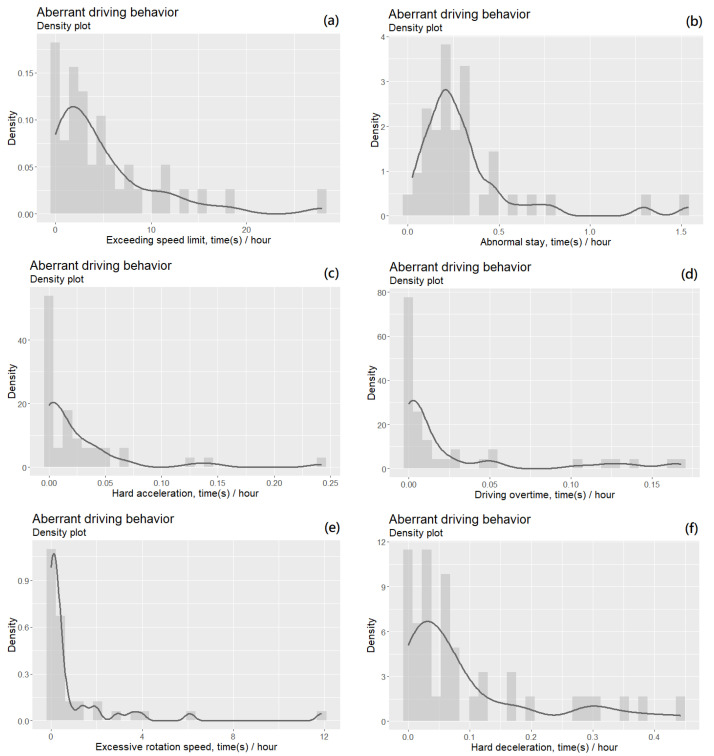
The density plots of aberrant driving behavior.

**Figure 2:**
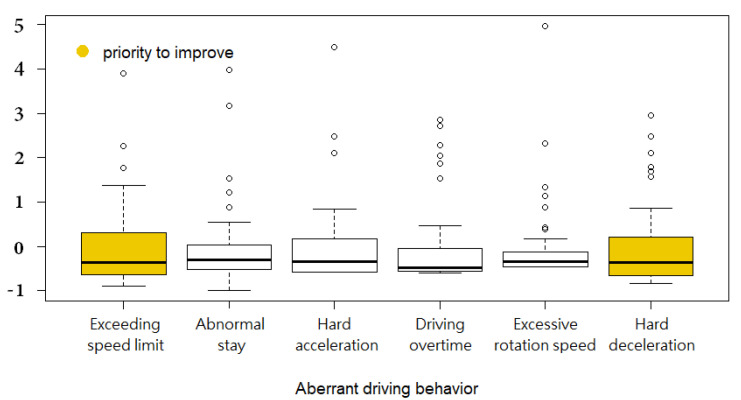
Boxplots of the aberrant driving behaviors.

Figure 1 shows the density plots of six aberrant driving behaviors. For discrete data, the sum of the areas of all rectangles in the density plots was always equal to 1; for continuous data, the area under the curve (definite integral) was also equal to 1. If the absolute value of the upper and lower bounds of *x* (data domain) in the probability density function was sufficiently small, then the density (*y* value) could be greater than 1. Figure 2c is an example of this, with *x* values between 0 and 0.25 and *y* values between 0 and 60.

If a set of data follows a normal distribution, then the *x* value corresponding to the peak of the plot represents the mean of the data. In our study, the peaks of occurrences of these six behaviors all fell to the left side of the plots, indicating that the distributions were positively skewed rather than having a normal distribution. However, we could still approximate the mean frequency of these behaviors from the plot. Based on the results from Figure 1a, drivers exceeded the speed limit approximately three times per hour on average. Moreover, we examined the statistical dispersion and central tendency of the data by observing the extent to which the distribution was stretched or squeezed.

Accordingly, we found that the density plots for exceeding the speed limit (Figure 1a) and hard deceleration (Figure 1f) had similar shapes and trends: A dispersed and positively skewed distribution. We, therefore, conjectured that the correlation between these two driving behaviors would be strong. The situation of Figure 1b, representing abnormal stay, was similar to that of Figure 1a,f, but its highest peak is further right. The other three behaviors in Figure 1c–e were more centralized and right-biased, especially excessive rotation speed; the majority of drivers displayed this behavior less than once an hour, and only a few drivers exceeded the average frequency.

Boxplots of the six aberrant driving behaviors are plotted in Figure 2. To compare these behaviors, all values have been standardized. From the risk management perspective, an ideal plot would contain a short box with short whiskers and few outliers, meaning that the data were centralized. For truck companies, aberrant driving behavior should ideally occur rarely, with minimal variance in the frequency of occurrence.

The results in Figure 2 are consistent with the findings obtained from the density plots. The dispersion of the occurrence of exceeding the speed limit and hard deceleration is high, indicating that these two driving behaviors had greater room for improvement. Therefore, if the truck company decided to take measures to reduce operational risk, these two behaviors should be addressed first. Additionally, although the distribution of excessive rotation speed was concentrated, this behavior cannot be neglected since the number of outliers was significant.

### 3.2. Correlation Analysis

Table 4 shows the result of the correlation analysis. Only a few correlation coefficients were higher than 0.7, and the values were not statistically significant at the 0.01 or 0.05 level. Therefore, all six aberrant driving behaviors will be considered for risk assessment.

Table 5 shows the results of the correlation analysis between personality traits and aberrant driving behaviors. We found that most correlation coefficients between personality traits and aberrant driving behaviors were lower than 0.35, and only a few correlation coefficients were significantly greater than 0.55. Based on the correlation analysis results, neuroticism was significantly related to exceeding the speed limit, abnormal stay, hard acceleration, excessive rotation speed, and hard deceleration. Conscientiousness was significantly related to driving overtime. Since most correlation coefficients were not significantly larger than 0.7, all five personality traits were incorporated in the model to predict aberrant driving behaviors and driving risk.

## 4. Aberrant Driving Behavior Classification and Driving Risk Classification

**Figure 3:**
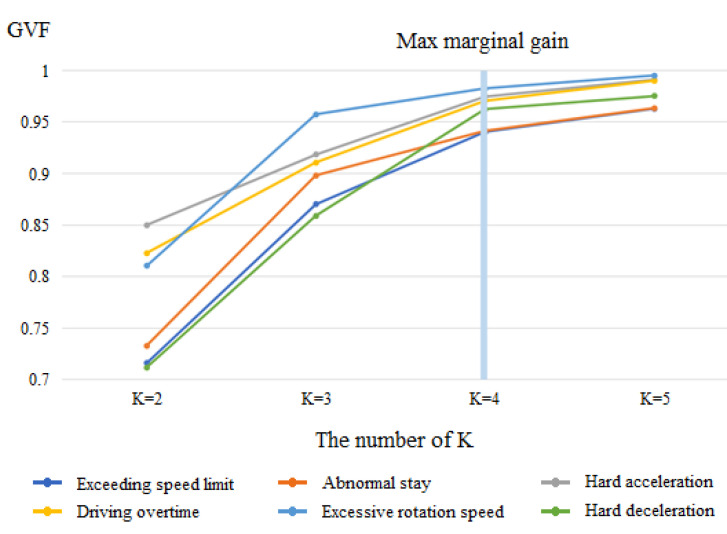
Elbow method to classify the aberrant driving behavior into subgroups.

Based on the results in Figure 3, the frequencies of aberrant driving behaviors among drivers were so different that it was difficult to directly use these data for model development. This study groups drivers into driving risk levels by driving behavior. A higher level means that the driver is at a higher risk of engaging in aberrant driving behavior.

For risk identification, the Jenks natural breaks optimization method [23] was applied to grade the aberrant driving behaviors and assign a driving risk index for each driver. The Jenks natural breaks optimization method is a data clustering method. It is an optimization process that finds the best way to arrange values into different classes (subgroups). It can be used for step-change detection in noisy data. Unlike other commonly used clustering methods (e.g., k-means, hierarchical clustering, etc.), this method is useful for subgrouping one-dimensional data and for analyzing data that are not evenly distributed. After the observations are classified, the grading results in the minimum intra-class deviation and the maximum inter-class deviation. The Jenks natural breaks optimization method [23] determines the best subgrouping result with the following iterative process:

The number of “k” classes (subgroups) is determined first.

Step 1. Calculate the “sum of squared deviations between classes (SDBC)”.
Step 2. Calculate the “sum of squared deviations from the array mean (SDAM)”.
Step 3. Subtract the SDBC from the SDAM, which equals the “sum of squared deviations from the class means (SDCM)”.
Step 4. After inspecting each of the SDBCs, move one unit from the class with the largest SDBC toward the class with the lowest SDBC.
Step 5. Repeat Steps 1 to 4 until the sum of the within-class deviations reaches a minimal value.
Step 6. Calculated the goodness of variance fit (GVF) by Equation (1), which ranges from 0 (worst fit) to 1 (perfect fit):

(1) $$\mathrm{GVF}=\frac{\mathrm{SDAM}-\mathrm{SDCM}}{\mathrm{SDAM}}=1-\frac{\mathrm{SDCM}}{\mathrm{SDAM}}$$

### 4.1. Aberrant Driving Behavior Classification

After calculating the GVF for each k classification of aberrant driving behavior, the elbow method was applied to determine the appropriate number of subgroups (k). The elbow method examines the percentage of variance that can be explained and determines a marginal gain that occurs by changing the value of k. We selected a set of k clusters thus that adding another cluster would not give much better modeling results for the dataset [24]. We tested the GVFs of k for 2, 3, 4, and 5 subgroups, as illustrated in Figure 3. When the k value increased from two to four subgroups, the GVF significantly increased. When the k value increased to five subgroups from four, GVF showed limited improvement for the model performance. Thus, the Jenks natural breaks optimization in this study was chosen to contain k = 4 subgroups.

The judgment boundaries and GVFs of each class level (subgroup) of aberrant driving behavior are shown in Table 6. The GVF for each aberrant driving behavior was higher than 0.7 and close to 1, meaning that the number of subgroups we chose was fairly fit for modeling purposes. A driver may have a high risk of being involved in a traffic crash when the driver is assigned to a higher class, such as Class 4.

Table 7 shows the numbers of drivers classified in each class level. For the six aberrant driving behaviors, more than 75% of drivers were classified as Class 1 or Class 2, and less than 8% of drivers were classified as Class 4.

### 4.2. Driving Risk Index Classification

**Figure 4:**
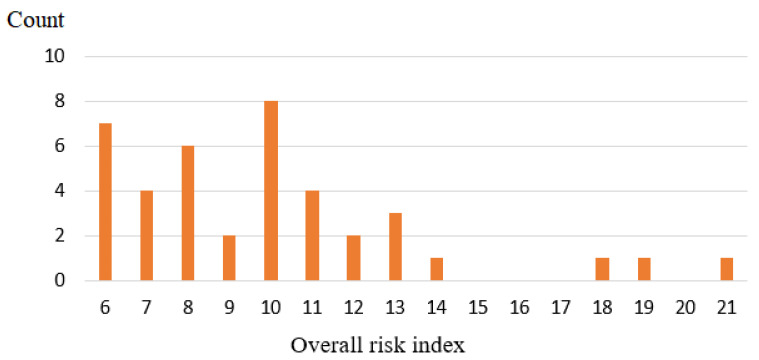
Distribution of overall driving risk indices.

Each driver’s driving risk index added up to the driver’s scores in each aberrant driving behavior. Every driver’s class level for each aberrant driving behavior was assigned as the driver’s score. The smaller the driver’s driving risk index, the lower the risk of the driver causing an accident. Figure 4 shows the distribution of the 40 drivers’ driving risk indices. The output range for the overall driving risk index was between 6 and 21.

The Jenks natural breaks optimization method and elbow method were also applied to classify the drivers by driving risk index values. We tested the GVF of k for 2, 3, 4, 5, 6, and 7 subgroups, as shown in Table 8. When the k value increased to six subgroups from five, the GVF showed limited improvement and only increased by 0.005 for the model performance. From the perspective of risk management, fewer subgroups lead to fewer differences between drivers, and the potentially high-risk drivers were not easy to be explored. Based on the driving risk index, we found that dividing the 40 drivers into 5 groups was appropriate.

Table 9 shows the classification results for drivers’ driving risk levels. Here, 7.5% of drivers were classified as level 5, and 15% of drivers were classified as level 4.

## 5. Aberrant Driving Behavior Class Prediction and Driving Risk Prediction

### 5.1. Model Development

**Figure 5:**
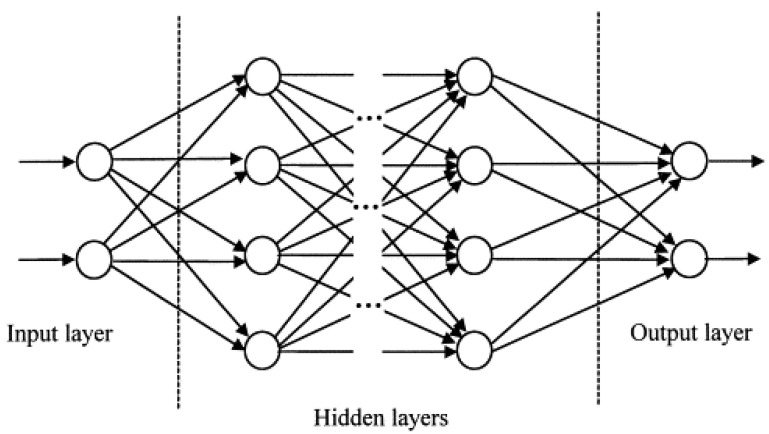
Schematic diagram of the ANN model.

After reviewing the literature, many previous studies have demonstrated that ANNs have the potential to predict traffic conditions on transportation issues accurately. ANNs can learn from events and make decisions through commenting on similar events, especially for the nonlinear relationship. Therefore, this research applied the ANN technique to explore a relational model between personality traits and the aberrant driving behavior class or driving risk. Figure 5 shows the preliminary structure of the ANN model, which usually consists of an input layer, several hidden layers, and an output layer.

When inputting variables into the network, the model calculates the weights from the input layer for the hidden layer. The transfer function in the hidden layer rescales the input data as inputs to the output layer. Since a discrepancy might occur between the estimated output and the actual class of each aberrant driving behavior for each driver, the weights were adjusted repeatedly by a suitable training method until the resulting error was stabilized and negligible. The function of aberrant driving behaviors inside the ANN model was determined after the completion of this training procedure [25]. This process is given by the following equation, where the output “*y*” is the estimated class of each aberrant driving behavior for each driver:

(2) $$y=g\left(\sum_{j}w_{hj}\times f\left(\sum_{i}w_{ih}\times x_{i}-\theta_{h}\right)-\theta_{j}\right)$$

where *y* is the output variable (the class of each aberrant driving behavior or driving risk for each driver, shown in Table 6); *i* are the elements at the input layer; *x_i_* are the input variables (five dimensions of personality traits for each driver, shown in Table 6); *h* is the elements at the hidden layer; *θ_h_* is the threshold values at the hidden layer; *w_ih_* are the weights between the input layer and hidden layer; *f* is the transfer function at the hidden layer; *θ_j_* is the threshold values at the output layer; *w_hj_* are the weights between the hidden layer and the output layer; and *g* is the transfer function at the output layer.

Table 10 shows the model inputs and outputs of ANN models in this study. All the models incorporated the average values for the five personality traits as the inputs (independent variables. Models 1, 2, 3, 4, 5, and 6 were developed to predict the class of each driver’s aberrant behavior, and these six models respectively incorporated one of the aberrant behaviors as the output. Models 7 and 8 were developed to predict the driver’s driving risk. Model 7 was used to predict the driver’s driving risk index, and Model 8 was used to predict the driver’s driving risk level, which was the classification of the drivers by groups according to their driving index.

### 5.2. Model Performance Assessment

The 40 drivers were divided into 2 groups. The data of 75% of the drivers in each class (level) were used for model training to explore the relational model between personality traits and the aberrant driving behavior class or driving risk. The data for the remaining drivers’ personality traits were entered into the developed models and were used to predict their aberrant driving behavior classes or driving risks.

To confirm the predictive ability of the model, we determined the accuracy (correct rate) for Models 1, 2, 3, 4, 5, 6, and 8 and the mean absolute percentage error (MAPE) and R^2^ for Model 7 to evaluate the performance of the predicted results. The advantage of MAPE is that it is not affected by the sample size and can accurately estimate the difference between the actual value and the estimated value [26]. The higher the accuracy, the smaller the MAPE and the closer *R*^2^ is to 1; this indicates that the learning ability and out of the model are better. The formulas used to calculate the predictive ability are given in the following equations, and the additional criteria for the MAPE are shown in Table 11:

(3) $$\mathrm{Accuracy}=\frac{\mathrm{the}\;\mathrm{number}\;\mathrm{of}\;\mathrm{samples}\;\mathrm{predicted}\;\mathrm{correctly}}{\mathrm{the}\;\mathrm{number}\;\mathrm{of}\;\mathrm{total}\;\mathrm{samples}}\times 100\%$$

(4) $$\mathrm{MAPE}=\frac{1}{n}\;\overset{n}{\underset{i=1}{\sum }}\left|\frac{observed_{i}-predicted_{i}}{observed_{i}}\right|\times 100\%$$

(5) $$R^{2}=\frac{{\left(\sum_{i=1}^{n}\left(obsvered_{i}-mean\;of\;obsvered\right)\left(predicted_{i}-mean\;of\;predicted\right)\right)}^{2}}{\sum_{i=1}^{n}{\left(obsvered_{i}-mean\;of\;obsvered\right)}^{2}\sum_{i=1}^{n}{\left(predicted_{i}-mean\;of\;predicted\right)}^{2}}$$

### 5.3. Results for the Evaluation of Aberrant Driving Behavior Class Prediction

To examine the proposed methodology, four experiments with stochastically sampling the training and testing data were conducted for each model. In each experiment, 75% of the drivers in each class were randomly selected for the training set, and the data of the remaining drivers were used for prediction and evaluation.

Table 12 presents the results of four experiments with ANN-based models for aberrant driving behavior class prediction. Based on the results in Table 12, the predictive accuracies for all six models were greater than 50%. The models for excessive rotation speed, exceeding the speed limit, hard deceleration, and hard acceleration made good predictions, with accuracies greater than 70% for most models. In particular, the accuracy of the model that predicted each driver’s class for excessive rotation speed was 90%. These results indicate that our model can reasonably predict the class of aberrant driving behaviors of truck drivers using personality traits. The model was especially useful in predicting the classes for excessive rotation speed, exceeding the speed limit, hard deceleration, and hard acceleration.

### 5.4. Evaluation of Results for Driving Risk Prediction

Table 13 shows the results of four experiments with the ANN-based models for driving risk prediction. Based on the results shown in Table 13, the MAPE values of Model 7 were between 7.9% and 11.7%, and the R^2^ values of Model 7 were between 0.79 and 0.87, which indicates a good forecasting level and highly accurate forecasting level, respectively. The predictive accuracies of Model 8 were higher than 70%. The evaluation results show that the proposed models can provide accurate prediction results that can be used to identify a driver’s driving risk index and level in advance.

## 6. Sensitivity Analysis

Sensitivity analysis can calculate the relative importance of a variable by changing the value of the input variable or removing the input variable from the model. This study applied sensitivity analysis to investigate the effect of personality traits on each aberrant driving behavior.

We use STATISTICA 13 software to do the sensitivity analysis process and to repeatedly replace the value of each input variable with the mean of the training sample. This value is then summed to the neural network repeatedly. The resulting network error is recorded and compared to the original error.

Table 14 shows a summary of the sensitivity analysis for this study. Since sensitivity analysis can only rate the importance of input variables, we also applied Spearman correlation to investigate the direction of correlation based on rank (nonlinear) correlation.

All the aberrant driving behaviors were affected by different personality traits to some degree. All the personality traits were positively correlated with speeding, the inference ratio of the Rank 1 factor was conscientiousness which had the greatest sensitivity (7.559), and the inference ratio of the rank 2 factor was agreeableness (4.486), which had the second greatest sensitivity. For abnormal stay and hard acceleration, all personality traits had similar sensitivities. All the personality traits were positively correlated with driving overtime. The inference ratio of the Rank 1 factor was conscientiousness, which had the greatest sensitivity (3.670). Similarly, all personality traits were positively correlated with excessive rotation speed; neuroticism had the greatest sensitivity (3.226) and was also significantly correlated for Spearman correlation (0.404 **). All the personality traits were positively correlated with hard deceleration; conscientiousness had the greatest sensitivity (5.102) and was also significantly correlated for Spearman correlation (0.399 *), while agreeableness had the second highest sensitivity (3.518). For both the driving risk index and level, the inference ratio of the Rank 1 factor was conscientiousness, which had the greatest sensitivity and was significant for the Spearman correlation.

In Table 14, which presents the sensitivity analysis results, the inference ratio of the Rank 1 factor was the first value listed for each prediction model. The Spearman correlations of the Rank 1 factors were not significant in most of our models.

To test the effects of the Rank 1 factor in Table 15 on the predictive performance of our models, we excluded the Rank 1 factor and used the other four factors for modeling. Table 15 compares the accuracies of the models before and after omitting the Rank 1 factor from the four experiments. After the conscientiousness factor was omitted from Models 1, 2, 4, 6, and 8, the predictive accuracy of the model decreased by 7.5%, 10%, 5%, 7.5%, and 7.5%, respectively. After the openness to experience factor was omitted from Model 3, the predictive accuracy of the model decreased by 7.5%. After the neuroticism factor was omitted from Model 5, the predictive accuracy of the model decreased by 7.5%. After the conscientiousness factor was excluded from Model 7, the MAPE value increased by 1.5%, and R^2^ decreased by 0.03. Based on the results in Table 15, Rank 1 factors were important for the proposed models and affected the predictive accuracy of the drivers’ aberrant driving behaviors and driving risk.

## 7. Conclusions

This study aimed to establish a driving risk evaluation mechanism based on aberrant driving behavior through risk index conversion and to further explore the correlation between personality traits, each aberrant driving behavior, and risk level classification. We collected information on the personality traits of truck drivers using a questionnaire, and we observed aberrant driving behaviors using a digital tachograph.

Through the Jenks natural breaks optimization method and the elbow method, 40 truck drivers were optimally classified into 4 aberrant driving behavior levels and 5 driving risk levels. Of these, 5% of drivers were at the highest aberrant driving behavior level and 7.5% of drivers were at the highest driving risk level.

To predict the drivers’ aberrant driving behavior level and driving risk level, this study applied an artificial neural network technique to develop eight prediction models, with five personality traits (extraversion, agreeableness, conscientiousness, neuroticism, and openness to experience) as the model inputs. According to the accuracy rate, MAPE, and R^2^, the proposed models showed good and stable model performance, especially in predicting the drivers’ class for the likelihood of excessive rotation speed, hard acceleration, and driving risk.

After sensitivity analysis, Models 1, 2, 4, 6, 7, and 8 indicated that drivers’ conscientiousness levels significantly related to the following aberrant driving behaviors: Exceeding the speed limit, abnormal stay, driving overtime, hard deceleration, driving risk index, and driving risk level; this human factor improved the prediction performance. Model 3 showed that drivers’ openness to experience is crucial for enhancing the prediction performance of hard acceleration. Model 5 indicated that a driver’s neuroticism is an important factor for increasing the prediction performance of excessive rotation speed.

### 7.1. Research Contribution in Empirical Application

By assessing the outputs of the model, this study shows that proposed models are a feasible way to predict the driving risk of truck drivers and to enhance safety management. With the proposed models, the predictive class for aberrant driving behavior and driving risk can be determined by plugging in a driver’s personality traits before or after employment. The manager of a transportation company can refer to the prediction results to plan the training program for each driver. Based on the sensitivity analysis results, conscientiousness is significantly correlated with speeding, hard deceleration, and driving risk index. The transportation company can arrange some training courses or speeches relating to conscientiousness for drivers to enhance their conscientiousness. After decreasing the aberrant driving behavior, the transportation company may reduce the probability of traffic crashes and further raise corporate integrity and reputation.

### 7.2. Research Limitation

The ANN model can preserve the information from all input variables to provide high-quality and accurate output results. However, the coefficient between each dependent variable and aberrant driving behavior cannot be directly presented in an ANN model. For this limitation, this research applies the sensitivity analysis to realize the significance of each variable to the aberrant driving behavior prediction.

To apply this research results to other types of professional drivers, a sufficient sample size would be desirable to represent driving behavior and corresponding personality traits.

## Figures and Tables

**Table 1:** Statistics on road traffic crashes in Taiwan, 2019. Source: [1].

Types of First Party	Sedan	Light Truck	Heavy Truck
Registered Vehicles (×10,000)	688.20	93.00	16.50
# Crashes	438.00	178.00	144.00
# Injuries	275.00	76.00	68.00
# Fatalities	449.00	189.00	147.00
Crash Rate Per 10,000 Vehicles	0.64	1.91	8.72
Injury Rate Per 10,000 Vehicles	0.40	0.82	4.12
Fatality Rate Per 10,000 Vehicles	0.65	2.03	8.90

**Table 2:** Personality questions based on the Big Five personality traits.

Dimensions and Questions	Average	S.D. ^+^
Extraversion	3.41	0.67
1. I am a person with leadership.	3.29	0.75
2. I like to stay in a lively place.	3.27	0.95
3. Others easily accept my opinion.	3.29	0.75
4. I am an active person.	3.56	0.74
5. I am an energetic person.	3.61	0.77
6. I like to chat with others.	3.44	0.78
Agreeableness	3.57	0.52
7. Most people that I know like me.	3.34	0.79
8. I enjoy working with others.	3.54	0.74
9. I am a person who always tries my best to help others	3.66	0.76
10. I am not a person who respects others. *	3.39	1.00
11. I get on well with my family or colleagues.	3.68	0.76
12. I consider other people’s positions.	3.71	0.64
13. I can accept different ideas.	3.66	0.69
14. I am a considerate person.	3.61	0.80
Conscientiousness	3.56	0.45
15. I am a stickler for routine.	3.76	0.62
16. I am a conscientious person.	3.73	0.59
17. I am a person who constantly pursues growth.	3.61	0.67
18. I often complete things on time.	3.66	0.57
19. I strive to be the best in everything I participate in.	3.46	0.71
20. I am a person who lacks planning skills.*	3.15	1.01
Neuroticism	2.77	0.44
21. I am a person who is easily upset.	2.95	0.97
22. I am a person with a stress tolerance.*	2.39	0.70
23. I often get angry at how others treat me.	3.07	0.82
24. I seldom feel lonely or depressed.*	2.56	0.78
25. I often feel nervous and jumpy.	2.78	0.91
26. I am a person who likes to be alone.	3.27	0.87
27. I am a person with emotional control.*	2.39	0.74
Openness to Experience	3.35	0.53
28. I am a person who always comes up with new methods.	3.39	0.80
29. I am a curious person.	3.41	0.81
30. I am a person who can think overall.	3.51	0.75
31. I am not a creative person. *	2.93	0.79
32. I am interested in thinking about the nature of the universe or the human environment.	3.49	0.81

* tables negative question, ^+^ Standard deviation. Source: [21,22].

**Table 3:** Statistics on the results of drivers’ aberrant driving behaviors.

	Total Number of Drivers	Average Number of Drivers	Minimum Number of Drivers	Maximum Number of Drivers
Exceeding speed limit	67,055	1676	0	7229
Abnormal stay	5712	143	7	559
Hard acceleration	426	11	0	63
Driving overtime	231	6	0	29
Excessive rotation speed	22,930	573	0	5131
Hard deceleration	1404	35	0	191

**Table 4:** Correlation analysis of aberrant driving behaviors.

	Aberrant Driving Behaviors					
	Exceeding Speed Limit	Abnormal Stay	Hard Acceleration	Driving Overtime	Excessive Rotation Speed	Hard Deceleration
Exceeding speed limit		0.569	0.540 *	0.003	0.552 **	0.757
Abnormal stay	0.569		0.609	−0.442	−0.563 *	0.610
Hard acceleration	0.540 *	0. 609		−0.280	0.735	0.700
Driving over-time	0.003	−0.442	−0.280		−0.289 *	−0.185
Excessive rotation speed	0.552 **	−0.563 *	0.735	−0.289 *		0.693 *
Hard deceleration	0.757	−0.610	0.700	−0.185	0.693 *	

**: Significant correlation is at 99% confidence level (2-tailed). *: Significant correlation is at 95% confidence level (2-tailed).

**Table 5:** Correlation analysis between personality traits and aberrant driving behaviors.

Personality Traits	Aberrant Driving Behaviors					
	Exceeding Speed Limit	Abnormal Stay	Hard Acceleration	Driving Overtime	Excessive Rotation Speed	Hard Deceleration
Extraversion	0.290	−0.132	0.210	0.209	−0.080	0.091
Agreeableness	−0.067	−0.125	0.062	0.319 *	−0.172	−0.031
Conscientiousness	0.018	−0.075	0.082	0.457 **	−0.141	0.053
Neuroticism	0.652 **	0.579 **	0.649 **	−0.287	0.835 **	0.691 **
Openness to experience	0.003	0.050	0.159	0.313 *	−0.071	0.093

**: Significant correlation is at 99% confidence level (2-tailed). *: Significant correlation is at 95% confidence level (2-tailed).

**Table 6:** The judgment boundaries of each class.

Aberrant Driving Behavior	Class 1		Class 2		Class 3		Class 4		GVF
	Lower	Upper	Lower	Upper	Lower	Upper	Lower	Upper	
Exceeding speed limit	0	778	1039	2060	2593	4334	5569	7231	0.94
Abnormal stay	7	79	135	212	242	344	404	559	0.94
Hard acceleration	0	7	10	20	33	39	57	63	0.97
Driving overtime	0	3	5	8	13	20	29	29	0.97
Excessive rotation speed	0	618	1702	2263	3378	3378	5108	5131	0.98
Hard deceleration	0	20	25	51	75	111	167	191	0.96

**Table 7:** Numbers of drivers in each class.

Aberrant Driving Behavior	Class 1	Class 2	Class 3	Class 4
Exceeding speed limit	15	16	6	3
Abnormal stay	22	8	7	3
Hard acceleration	29	4	5	2
Driving overtime	22	10	6	2
Excessive rotation speed	34	3	1	2
Hard deceleration	20	14	4	2

**Table 8:** Elbow method to classify the driving risk index into subgroups.

Number of Groups	k = 2	k = 3	k = 4	k = 5	k = 6	k = 7
GVF	0.617	0.887	0.947	0.971	0.976	0.987

**Table 9:** Classification results for drivers’ driving risk levels.

Level	Threshold		Number of Drivers	%
	Lower	Upper		
Class 1	6	7	11	27.5
Class 2	8	9	8	20.0
Class 3	10	11	12	30.0
Class 4	12	14	6	15.0
Class 5	18	21	3	7.5

**Table 10:** Inputs and outputs of the ANN models.

Prediction	Models	Input layer	
		Inputs (independent variables)	Contents
Both Aberrant driving behavior and Driving risk	All Models	*x*_1_: Extraversion	5: Strongly agree, 4: Agree, 3: Undecided, 2: Disagree, 1: Strongly disagree
		*x*_2_: Agreeableness	
		*x*_3_: Conscientiousness	
		*x*_4_: Neuroticism	
		*x*_5_: Openness to experience	
**Prediction**	**Models**	**Output layer**	
		Output (dependent variable)	Contents
Aberrant driving behavior	Model 1	Exceeding the speed limit	1: Class 1, 2: Class 2, 3: Class 3, 4: Class 4 (High aberrant)
	Model 2	Abnormal stay	
	Model 3	Hard acceleration	
	Model 4	Driving overtime	
	Model 5	Excessive rotation speed	
	Model 6	Hard deceleration	
Driving risk	Model 7	Driving risk index	Integer (Min: 6, Max: 21)
	Model 8	Driving risk level	1: Class 1, 2: Class 2, 3: Class 3, 4: Class 4, 5: Class 5 (High risk)

**Table 11:** Interpretation of typical MAPE values.

MAPE Values	Interpretation
Less than 10%	Highly accurate forecasting
11% to 20%	Good forecasting
21% to 50%	Reasonable forecasting
51% or more	Inaccurate forecasting

Source: [26].

**Table 12:** Predictive accuracy of the proposed models for aberrant driving behavior.

		Experiment	1	2	3	4	Average
	Predicted aberrant driving behavior						
Model 1	Exceeding the speed limit		80%	80%	70%	60%	72.5%
Model 2	Abnormal stay		50%	60%	60%	60%	57.5%
Model 3	Hard acceleration		60%	80%	70%	70%	70.0%
Model 4	Driving overtime		50%	60%	70%	70%	62.5%
Model 5	Excessive rotation speed		80%	80%	90%	90%	85.0%
Model 6	Hard deceleration		70%	80%	70%	80%	75.0%

**Table 13:** Predicted performance of the proposed models for driving risk.

		Experiment	1	2	3	4	Average
	Predicted driving risk						
Model 7	Driving risk index	MAPE	11.7%	11.1%	7.9%	10.2%	10.2%
		R^2^	0.79	0.87	0.83	0.86	0.84
Model 8	Driving risk level	Accuracy	60%	90%	70%	70%	73%

**Table 14:** Sensitivity analysis of personality traits for proposed models.

		Personality Traits				
		Extraversion	Agreeableness	Conscientiousness	Neuroticism	Openness to Experience
Model 1	Average ratio	2.8572	4.4864	7.5591	1.8874	2.9228
	(Spearman correlation)	(0.240)	(0.129)	(0.281)	(0.210)	(0.162)
	Rank	4	2	1	5	3
Model 2	Average ratio	0.9991	0.9992	1.0010	0.9999	1.0007
	(Spearman correlation)	(−0.094)	(−0.004 **)	(0.145)	(0.095)	(0.262)
	Rank	5	4	1	3	2
Model 3	Average ratio	0.9995	0.9972	0.9996	0.9993	1.0009
	(Spearman correlation)	(0.259)	(0.232)	(0.364 *)	(−0.251)	(0.187)
	Rank	3	5	2	4	1
Model 4	Average ratio	1.1559	1.9549	3.6701	1.1174	1.2325
	(Spearman correlation)	(0.404 **)	(0.178)	(0.218)	(0.393 *)	(0.325 *)
	Rank	4	2	1	5	3
Model 5	Average ratio	1.1133	1.5301	1.2090	3.2265	1.0910
	(Spearman correlation)	(0.258)	(0.131)	(0.135)	(0.404 **)	(0.260)
	Rank	4	2	3	1	5
Model 6	Average ratio	1.2190	3.5187	5.1026	1.5284	1.5871
	(Spearman correlation)	(0.379 *)	(0.196)	(0.399 *)	(0.289)	(0.325 *)
	Rank	5	2	1	4	3
Model 7	Average ratio	1.0170	2.2137	3.5308	2.6199	1.0808
	(Spearman correlation)	(0.244)	(0.160)	(0.385 *)	(0.380 *)	(0.345 *)
	Rank	5	3	1	2	4
Model 8	Average ratio	1.1102	1.3415	1.7101	1.1638	1.0625
	(Spearman correlation)	(0.224)	(0.156)	(0.381 *)	(0.390 *)	(0.354 *)
	Rank	4	2	1	3	5

**: Significant correlation is at 99% confidence level (2-tailed). *: Significant correlation is at 95% confidence level (2-tailed).

**Table 15:** Predicted performance comparison of the proposed models.

		Experiment	1	2	3	4	Average
	Personality traits in model inputs						
Model 1	All five personality traits	Accuracy	80%	80%	70%	60%	72.5%
	Excluding the “Conscientiousness” factor	Accuracy	70%	70%	70%	50%	65.0%
	Performance difference after excluding the “Conscientiousness” factor		−10%	−10%	0%	−10%	−7.5%
Model 2	All five personality traits	Accuracy	50%	60%	60%	60%	57.5%
	Excluding the “Conscientiousness” factor	Accuracy	50%	40%	50%	50%	47.5%
	Performance difference after excluding the “Conscientiousness” factor		0%	−20%	−10%	−10%	−10.0%
Model 3	All five personality traits	Accuracy	60%	80%	70%	70%	70.0%
	Excluding the “Openness to Experience” factor	Accuracy	60%	60%	70%	60%	62.5%
	Performance difference after excluding the “Openness to Experience” factor		0%	−20%	0%	−10%	−7.5%
Model 4	All five personality traits	Accuracy	50%	60%	70%	70%	62.5%
	Excluding the “Conscientiousness” factor	Accuracy	40%	50%	70%	70%	57.5%
	Performance difference after excluding the “Conscientiousness” factor		−10%	−10%	0%	0%	−5.0%
Model 5	All five personality traits	Accuracy	80%	80%	90%	90%	85.0%
	Excluding the “Neuroticism” factor	Accuracy	80%	70%	90%	70%	77.5%
	Performance difference after excluding the “Neuroticism” factor		0%	−10%	0%	−20%	−7.5%
Model 6	All five personality traits	Accuracy	70%	80%	70%	80%	75.0%
	Excluding the “Conscientiousness” factor	Accuracy	60%	80%	60%	70%	67.5%
	Performance difference after excluding the “Conscientiousness” factor		−10%	0%	−10%	−10%	−7.5%
Model 7	All five personality traits	MAPE	11.7%	11.1%	7.9%	10.2%	10.2%
	Excluding the “Conscientiousness” factor	MAPE	11.9%	13.5%	8.7%	12.9%	11.7%
	Performance difference after excluding the “Conscientiousness” factor		0.2%	2.4%	0.8%	2.7%	1.5%
Model 7	All five personality traits	R^2^	0.79	0.87	0.83	0.86	0.84
	Excluding the “Conscientiousness” factor	R^2^	0.78	0.84	0.82	0.81	0.81
	Performance difference after excluding the “Conscientiousness” factor		−0.01	−0.03	−0.01	−0.05	−0.03
Model 8	All five personality traits	Accuracy	60%	90%	70%	70%	73%
	Excluding the “Conscientiousness” factor	Accuracy	60%	70%	70%	60%	65%
	Performance difference after excluding the “Conscientiousness” factor		0%	−20%	0%	−10%	−7.5%

## Data Availability

Data sharing is not applicable to this article.
